# Editorial: Mechanisms driving drug resistance in tuberculosis and malaria: genetic, environmental, and evolutionary insights

**DOI:** 10.3389/fcimb.2025.1692516

**Published:** 2025-09-22

**Authors:** Ambuj Kumar Kushwaha, Abhisheka Bansal, Gunjan Arora, Divya Kushwaha

**Affiliations:** ^1^ School of Biotechnology, Institute of Science, Banaras Hindu University, Varanasi, India; ^2^ Cellular and Molecular Parasitology Laboratory, School of Life Sciences, Jawaharlal Nehru University, New Delhi, India; ^3^ Section of Infectious Diseases, Department of Internal Medicine, Yale School of Medicine, New Haven, CT, United States; ^4^ Department of Chemistry, Mahila Mahavidyalaya (MMV), Banaras Hindu University, Varanasi, India

**Keywords:** malaria, tuberculosis, plasmodium, mycobacterium, drug resistance, infectious diseases, enterobacter, disinfectant

Tuberculosis (TB) and malaria remain two of the most formidable infectious diseases, continuing to exert a disproportionate toll on global health despite decades of scientific and medical advances. Pathogenic *Mycobacterium* species are responsible for major human diseases such as TB, leprosy, and Buruli ulcer (Walsh et al., 2010; Arora et al., 2021). Of these, TB caused by *Mycobacterium tuberculosis* (*Mtb*) remains the leading cause of death from a single infectious agent, owing partly to its capacity for dormancy (Kushwaha and Bhushan, 2020). According to the WHO Global Tuberculosis Report 2024, cases increased from 10.1 million in 2020 to 10.8 million in 2023, with India, Indonesia, China, the Philippines, and Pakistan together accounting for more than half of the global burden (World Health, 2024a). Progress remains insufficient, with an incidence decline of only 8.3% since 2015, far below the WHO End TB Strategy’s 2025 target.

Malaria, caused by protozoan parasites of the genus *Plasmodium*, poses a comparable global health challenge, with 11 million additional cases in 2023 compared to 2022. Most cases are concentrated in sub-Saharan Africa, and global incidence remains threefold higher than the WHO Global Technical Strategy 2025 benchmark (World Health, 2024b).

Though caused by distinct pathogens, TB and malaria share critical obstacles: drug resistance, limited vaccine efficacy, treatment non-adherence, and frequent co-infections in endemic regions (Duffey et al., 2024; Farhat et al., 2024). These challenges emphasize the urgent need to advance host-directed therapies, develop novel drugs with well-defined mechanisms of action, identify new drug targets, and enhance vaccines and diagnostic tools (Basore et al., 2015; Kushwaha et al., 2018; Matteucci et al., 2022).

This Frontiers in Cellular and Infection Microbiology Research Topic, *“Mechanisms Driving Drug Resistance in Tuberculosis and Malaria: Genetic, Environmental, and Evolutionary Insights”*, brings together seven original articles that highlight microbial adaptation, resistance mechanisms, and diagnostic innovations, offering new perspectives to confront these enduring challenges. The major findings and contributions of these studies are outlined in the following sections.

## UHPLC-HRMS approaches to plasma biomarkers for tuberculosis detection

It is estimated that approximately 25% of the global population is infected with *Mycobacterium tuberculosis*, although only a subset develops active disease (Goletti et al., 2025). Conventional microbiological methods suffer from low sensitivity, while immunological assays are limited in reliably distinguishing latent from active TB, emphasizing the urgent need for innovative diagnostic approaches (MacLean et al., 2020). Biomarkers in body fluids represent a promising avenue (Maji et al., 2018). In this context, Sun et al. profiled plasma metabolites in 72 TB patients and 78 controls, with validation in an independent cohort. Using high-resolution mass spectrometry and machine learning, they identified 22 lipids altered in TB patients, alongside seven key metabolites, of which Angiotensin IV showed high diagnostic accuracy. These findings highlight lipid and metabolite alterations as potential biomarkers, though further validation is essential. Integration of metabolomics with AI approaches could transform diagnostics for TB and other infectious diseases.

## Targeting *Mycobacterium tuberculosis* via vitamin C–primed Vγ9Vδ2 T-cells

Although there are several vaccine candidates in clinical trials, the limitations of the current BCG vaccine highlight the need for new strategies against TB (Cobelens et al., 2022; Wilson et al., 2023; Starshinova et al., 2025). In this study, Liu et al., highlight the importance of the Vγ9Vδ2 T-cell subset in inhibiting *Mtb*. Researchers expanded Vγ9Vδ2 T cells *in vitro* using (E)-4-hydroxy-3-methyl-but-2-enyl pyrophosphate (HMBPP), vitamin C (VC), and rIL-2, and observed a 40–60% inhibition of the virulent *Mtb* H37Rv strain. Notably, VC enhanced the expansion of HMBPP-primed cells, which restricted intracellular *Mtb* growth through upregulation of TNF-α and IFN-γ. These findings suggest a promising immunotherapeutic approach, offering new directions for TB control and future vaccine development.

## Epidemiological insights into drug-resistant osteoarticular TB in south central China

Though pulmonary TB is well-studied, osteoarticular tuberculosis (OATB), a challenging, underdiagnosed form of non-pulmonary TB, remains poorly characterized, particularly in terms of drug resistance. In this study, Fang et al. analyzed nine years of retrospective data from 269 culture-confirmed OATB cases at Hunan Chest Hospital, China. The study revealed a high resistance burden, with nearly one-third of isolates resistant to at least one first-line drug and 14% identified as multidrug-resistant. Resistance was significantly associated with male sex, farming occupation, and age groups 20–29 and 60–69 years. These findings underscore the urgent need for rapid molecular drug susceptibility testing and region-specific treatment protocols, advocating for targeted public health strategies to curb resistance and improve outcomes in OATB.

## DosR-driven adaptation of *Mycobacterium avium* to hypoxia

Non-tuberculous mycobacteria (NTM) are increasingly recognized as important pathogens causing pulmonary infections, with *Mycobacterium avium* complex (MAC) and *Mycobacterium abscessus* being the most clinically relevant species (Dahl et al., 2022). DosRS, a key regulatory system in *Mtb*, enables adaptation to hypoxic stress and persistence within host environments (Kendall et al., 2004). To evaluate its role in *M. avium*, Belardinelli et al. exposed bacteria to microaerophilic conditions and assessed transcriptomic changes using RNA-Seq. Sixteen stress response genes were significantly upregulated, but this induction was largely abolished in a *dosRS*-deficient mutant, which also showed growth defects under acidic conditions. Interestingly, DosRS deletion did not affect biofilm formation or resistance to rifampicin and clarithromycin, though mutants exhibited increased susceptibility to nitrofuran derivatives. In contrast, *M. abscessus* lacking DosRS showed altered biofilm and antibiotic tolerance, indicating species-specific regulatory roles. Taken together, these findings indicate that DosRS contributes to hypoxic survival in *M. avium* but plays a less prominent role in antibiotic tolerance compared to other NTMs.

## Integrons as drivers of antibiotic resistance in *Enterobacter cloacae*

The emergence of multidrug-resistant (MDR) bacteria alters pathogenesis, worsens clinical outcomes, and increases mortality (Kumar and Chordia, 2017; Arora et al., 2021). Among them, *Enterobacter cloacae* complex (ECC) is a major opportunistic pathogen causing pneumonia, urinary tract infections (UTI), septicemia, and death in hospitalized or immunocompromised patients (Sanders and Sanders, 1997; Liu et al., 2024). Rising resistance in ECC, particularly to β-lactams, has become a critical healthcare concern (Liu et al., 2024). The spread of resistance is facilitated by integrons, genetic elements that capture and disseminate resistance genes *via* horizontal gene transfer (Sabbagh et al., 2021). In this article, Qiu et al. examined 80 *E. cloacae* isolates from UTI patients in China, detecting Class 1 integrons in 31 (38.8%) strains and Class 2 integrons in 5 (6.3%) strains, with none harboring Class 3 integrons. Integron-positive strains showed significantly higher resistance to multiple antibiotics. These findings highlight integrons as key drivers of MDR *E. cloacae*, underscoring their role in surveillance and infection control.

## Adaptation of bacterial isolates to disinfectants in healthcare settings

The emergence of antimicrobial resistance (AMR) is a global challenge that increases the risk of infections in places presumed to be safe, including hospitals. The study by Rakshit et al. examined resistance to disinfectants such as benzalkonium chloride, sodium hypochlorite, glutaraldehyde, and chlorhexidine (CHX). Four resistant isolates were identified, two *Klebsiella pneumoniae* and two *Pseudomonas aeruginosa*, which survived exposure to CHX-based disinfectants and displayed elevated MIC and MBC values against glutaraldehyde and sodium hypochlorite. All isolates produced strong biofilms with limited reduction even at higher disinfectant concentrations. Whole-genome sequencing revealed multiple resistance determinants, including *bla*_DIM-1_, disinfectant-resistance, and efflux pump genes. These findings highlight adaptive mechanisms that facilitate bacterial persistence in disinfectant-rich hospital environments, complicating infection control.

## Quiescin sulfhydryl oxidase as a transmission-blocking antigen in *Plasmodium vivax*

Malaria parasites exhibit resistance or delayed clearance to all available drugs, including frontline artemisinin. Current vaccines provide only limited protection and are restricted to *Plasmodium falciparum* malaria (Baird, 2013; Datoo et al., 2021; Rosenthal et al., 2024; White and Chotivanich, 2024; Yamamoto et al., 2025). *P. falciparum* and *P. vivax* account for almost all global malaria burden, with *P. vivax* causing chronic and relapsing infections due to dormant liver hypnozoites and showing increasing prevalence because of its high transmissibility. Transmission-blocking vaccines (TBVs), which target parasite proteins that play essential roles in sexual stage development within mosquitoes, offer a promising control strategy. Antibodies against such proteins, ingested during mosquito feeding, disrupt parasite maturation and lower transmission rates in endemic regions. Established TBV candidates include Pfs230, Pfs48/45, and Pfs25 (Duffy, 2021; Tachibana et al., 2025). In this study, Zheng et al. identified Quiescin sulfhydryl oxidase (QSOX) as a potential TBV target in *P. vivax*. Anti-PvQSOX antibodies reduced ookinete and oocyst formation in mosquito infections with transgenic *P. berghei* and *P. vivax* field isolates, supporting further evaluation of PvQSOX as part of TBV strategies.

## Conclusion and future directions

The genetic adaptation of pathogens to antibiotics and therapeutics poses a major challenge to disease control and public health. Addressing this requires a multidisciplinary approach that includes discovery of novel drug targets and vaccines, systematic epidemiological studies, and understanding the evolution and distribution of resistance phenotypes. Genomic surveillance and advanced molecular diagnostics are critical for early detection and targeted interventions. Looking ahead, strengthening infrastructure, capacity building, and fostering international collaboration will be essential to combat emerging resistance. Sustained investment in these areas will help mitigate the global burden of antimicrobial resistance and ensure effective long-term disease management.
